# The Spanish gut microbiome reveals links between microorganisms and Mediterranean diet

**DOI:** 10.1038/s41598-021-01002-1

**Published:** 2021-11-10

**Authors:** Adriel Latorre-Pérez, Marta Hernández, Jose Ramón Iglesias, Javier Morán, Javier Pascual, Manuel Porcar, Cristina Vilanova, Luis Collado

**Affiliations:** 1 Darwin Bioprospecting Excellence S.L., Paterna, Spain; 2 Instituto Central Lechera Asturiana para la Nutrición Personalizada (ICLANP), Siero, Spain; 3 Instituto de Innovación Alimentaria, Universidad Católica de Murcia, Murcia, Spain; 4 Institute for Integrative Systems Biology (I2SysBio), University of València-CSIC, Paterna, Spain; 5 Department of Medicine, Complutense University of Madrid, Madrid, Spain

**Keywords:** Microbiology, Bacteria, Microbial communities, Metagenomics

## Abstract

Despite the increasing evidence of links between human gut and health, the number of gut microbiomes that have been studied to date at a country level are surprisingly low. Mediterranean countries, including some of the most long-lived and healthy countries in the world, have not been considered so far in those studies at a large scale. The main objective of this work is to characterize the gut microbiome of a healthy adult population of a Mediterranean, paradigmatically healthy country: Spain. Stool samples from 530 healthy volunteers were collected, total metagenomic DNA extracted, and the microbial profiles determined through 16S rRNA metataxonomic sequencing. Our results confirm the associations between several microbial markers and different variables, including sex, age, BMI and diet choices, and bring new insights into the relationship between microbiome and diet in the Spanish population. Remarkably, some of the associations found, such as the decrease of *Faecalibacterium* with age or the link of *Flavonifractor* with less healthy dietary habits, have been barely noticed in other large-scale cohorts. On the other hand, a range of links between microorganisms, diet, and lifestyle coincide with those reported in other populations, thus increasing the robustness of such associations and confirming the importance of these microbial markers across different countries. Overall, this study describes the Spanish “normal” microbiome, providing a solid baseline for future studies investigating the effects of gut microbiome composition and deviations in the adherence to the Mediterranean diet.

## Introduction

Human gut is one of the most diverse ecosystems on Earth. As a result of millions of years of co-evolution, gut microorganisms perform essential activities for human health and nutrition, from the digestion of vegetal fiber^1^ to the regulation of complex signalling pathways acting beyond our gut^2,3^. Since the development of metagenomic sequencing techniques, the human gut microbiome has been a recurrent object of study. In 2007, The Human Microbiome Project (HMP) was launched with two main objectives: (1) understanding the dimension of the microbial communities associated to the human body, regarding variability among individuals; and (2) shedding light on the interplay between gut microbiota and a range of diseases^4,5^. To date, nearly 6.000 gut microbiome samples (out of more than 31.000 corresponding to different body sites) have been analyzed in the framework of the HMP. These datasets originate from individuals of different sex, age, culture, geographic location, and health status, which implies multiple potential connections between the composition of the gut microbiome and a range of health issues and diseases^6,7^. In order to shed light on those correlations, the microbiome profiles of different cohorts (usually, healthy vs diseased) are often compared, and differences involving single microorganisms^8,9^, microbial consortia^10^, or dynamic behaviors of the community are identified^11^.

The definition of a “normal” (or “healthy”) microbiome is crucial to understand how this microbiome is altered as a consequence of any factor. However, the systematic analysis of the microbiome of healthy individuals has only been addressed by a few studies, and the very concept of “normal” microbiome is still controversial^12,13^. Since a range of factors associated to climate, geography, and culture are known to influence the gut microbiome^14–16^, the precise definition of a normal microbiome should be restricted to a given population, of the same region/country and with similar diet and lifestyle habits. Moreover, and in order to assess the associations of microorganisms with specific phenotypic or environmental factors (e.g., age, sex, diet, disease states, etc.), it is necessary to know the natural variability in healthy individuals through the study of large cohorts, since many traits are multifactorial and caused by small effects from many variants^13,17^. To date, only a few large (over 500 people) cohort-based human faecal microbiome studies have been conducted, including English, Dutch, Belgian, German, Israeli, North American, and South African cohorts^13,17–21^. These studies have identified microbial markers associated with phenotypic traits^13,17–19^, pathological processes, medicine consumption^21^, and lifestyle^20^, among other factors.

With a population of more than 47 million citizens, Spain represents a particular case of study. From a geographical point of view, the Spanish territory includes the peninsular region, the Balearic and Canary Islands, and the cities of Ceuta and Melilla (located on the northwest coast of Africa). Regarding climate, Spain is the most diverse country in Europe, including regions with oceanic, warm Mediterranean, hot Mediterranean, and arid climates in the peninsula, and a tropical climate in the Canary Islands. One of the main traits of the Spanish culture is the Mediterranean diet, characterized by the prevalent uptake of vegetables, fruits, legumes, beans, cereals, fish, and unsaturated fats such as olive oil. The health benefits of this type of diet are well known^22,23^, and its impact on the gut microbiome has been addressed in recent works^24,25^. This, along with other cultural and social aspects, is one of the main reasons why Spain is considered the country with the highest lifespan^26^ and life quality^27^.

The objective of the present work is to shed light on the "normal" microbiome of the Spanish population, as a particular case of Mediterranean countries, in which gut microbiomes, at a epidemiological level, have surprisingly not been studied so far in a comprehensive way. In order to fill this gap, a representative cohort of individuals was selected according to demographic criteria (age, sex, and geographic location), and their stool microbiome was analyzed through 16S rRNA metataxonomic sequencing following the HMP standard guidelines. Our results depict the diversity and variability of the Spanish microbiome regarding sex, geography, and age range of healthy individuals, and confirm or unveil new microbiome-health correlations among particular microbial markers and a range of diet variables, most of which involve the consumption of food associated to the Mediterranean diet.

## Results

### Dataset description

A total of 530 individuals (267 females and 263 males) across the Spanish territory (including the Canary and Balearic Islands, Ceuta and Melilla) joined the study (Supplementary Table 1). The sample was designed to be representative of the Spanish population in terms of sex, age and geographical distribution. All the samples were associated to basic metadata of the donor (age range, sex and region), while 528 participants provided extended metadata (weight, height, eating habits, medical conditions, etc.) via online questionnaires (Supplementary Table 2). Microbiome sequencing resulted in 36,441,101 joined reads after quality filtering and chimera removal, yielding a median of 71,402 reads per sample (Q1 = 41,271, Q3 = 93,216). Rarefaction curves indicated that all the samples reached the asymptote (Supplementary Fig. 1).

### The Spanish gut microbiome

A total of 25,764 different ASVs were detected in the entire dataset. At the phylum level, the average Spanish gut microbiome was dominated by *Firmicutes* (~ 53.9%) and *Bacteroidota* (or *Bacteroidetes*) (~ 37.2%), followed by *Proteobacteria* (~ 5%)*, Verrucomicrobiota* (or *Verrucomicrobia*) (~ 1.8%), and *Actinobacteriota* (or *Actinobacteria*) (~ 0.9%). The average *Firmicutes*/ *Bacteroidota* (F/B) ratio was 1.78 (CI 1.99–1.57).

The dominant genera were *Bacteroides* (~ 18.4%) and *Faecalibacterium* (~ 12.5%), followed by *Prevotella* (6.7%), *Alistipes* (~ 3.4%), and *Oscillospiraceae* UCG-002 (~ 2.3%). An average of 100 ± 18 different genera (out of 447 globally detected), and 8 ± 2 different phyla (out of 21) were detected in each individual sample. The average richness and Shannon indices at the ASV level were 283 ± 77 and 4.3 ± 0.4, respectively.

**Figure 1:**
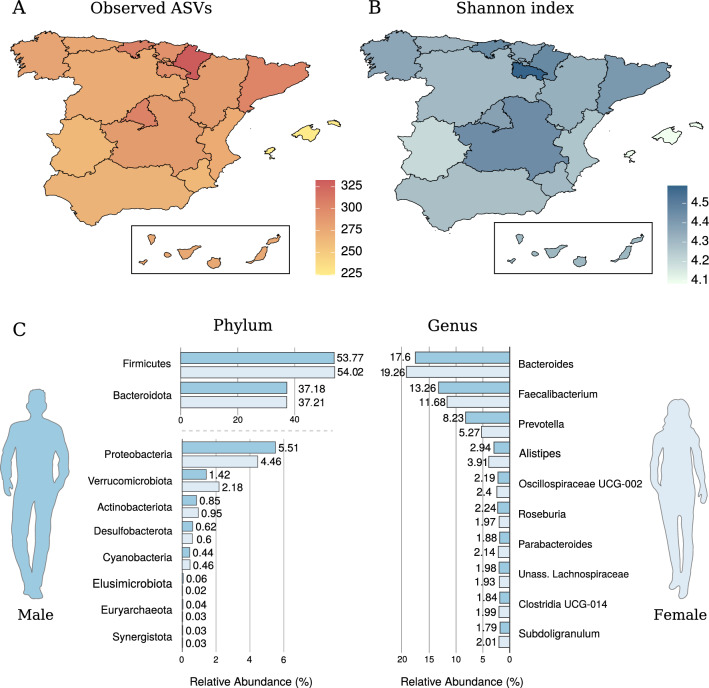
Diversity indices (**A**: Richness or Observed ASVs; **B**: Shannon) across the Spanish territory and (**C**) distribution of the main taxa (phylum and genus level) in the gut microbiome of male and female individuals. Maps were created with rdgal (v. 1.5-16; https://cran.r-project.org/web/packages/rgdal/index.html) and ggplot2 (v. 3.3.1; https://ggplot2.tidyverse.org/).

The Balearic Islands presented the lowest average values for alpha diversity metrics (richness: 224.5; Shannon: 4.1), while Navarra and La Rioja regions (Northern Spain) showed the highest richness (332.3; Fig. 1A) and Shannon values (4.6; Fig. 1B), respectively. It is worth highlighting that the number of participants recruited for each Spanish region was proportional to the population of this region with respect to the total population of Spain (Supplementary Table 1). As this could result in a bias towards higher or lower microbial diversity in the most or least represented regions in the cohort, this effect was further investigated, and no significant association was found between the number of individuals and the average alpha diversity metrics of each region (*p*-value > 0.05; Spearman’s rank correlation) (Supplementary Fig. 2). To study the region-specific patterns of microbial diversity, the cohort was divided in three main categories: Mediterranean regions (Catalonia, Valencian Community, Region of Murcia and Andalusia), islands (Canary and Balearic Islands), and rest of the Spanish mainland. No significant differences were found across these regions in terms of alpha diversity. Results of the pairwise comparisons of the individual regions (i.e., Spanish Autonomous Regions) were not significant either (FDR-adjusted *p*-value > 0.05; Wilcoxon rank-sum test; Supplementary Table 3). Interestingly, gut microbial profiles from people living in islands differed from those profiles obtained from participants who resided in mainland Spain. In particular, the phylum *Patescibacteria* and twelve other genera were significantly over- or under-represented when comparing island and mainland profiles (*p*-value < 0.05; DESeq2 test) (Supplementary Table 4). In contrast, no significant differences were found between Mediterranean regions and the rest of the Spanish mainland.

**Figure 2:**
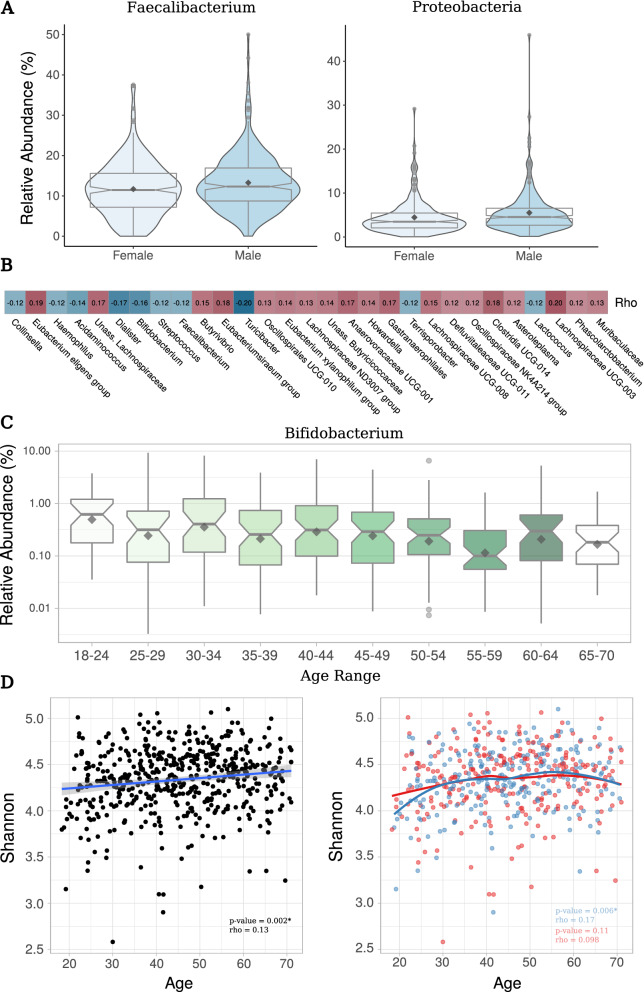
Associations of particular markers with sex and age. (**A**) Relative abundance of *Faecalibacterium* and *Proteobacteria* in male and female individuals. (**B**) Genera showing a significant Spearman’s rank correlation (FDR-adjusted *p*-value < 0.05) with age. Rho = Spearman's rank correlation coefficient. (**C**) Relative abundance of *Bifidobacterium* in different age groups. (**D**) Shannon index vs. age considering the whole cohort (left) (linear model “lm” used as smoothing method) or separating by sex (right) (local polynomial regression fitting model “loess” used as smoothing method). Rho and *p*-values resulting from Spearman’s rank correlation are shown. Black = both females and males; Red = females; Blue = males.

Gut microbiomes of males and females were markedly similar (Fig. 1C). Nevertheless, the abundance of the phylum *Proteobacteria* and the genus *Faecalibacterium* were significantly higher in males than in females (*p*-value < 0.05; DESeq2 test) (Fig. 2A). There were eight additional genera whose abundances differed between these two groups, including *Oscillibacter* and *Anaerostipes* (Supplementary Table 5). No significant differences between genders were found in terms of alpha diversity (*p*-value > 0.05; Wilcoxon rank-sum test).

A relatively high number of microbial markers were associated with age. Specifically, age significantly correlated with one phylum (*Cyanobacteria*) and 29 different genera (Fig. 2B and Supplementary Table 6) (*p*-value < 0.05; Spearman’s rank correlation). Eight of these genera (*Faecalibacterium, Clostridia* UCG-014, *Phascolarctobacterium, Eubacterium eligens* group, *Dialister, Eubacterium siraeum* group, an unassigned *Muribaculaceae*, and an unassigned *Lachnospiraceae*) were among the top 30 taxa with the highest average relative abundance (rel. abund. > 0.7%). Remarkably, *Bifidobacterium* relative abundance also decreased with age (Fig. 2C). Both alpha diversity metrics studied (richness & Shannon; ASV-level) were also age-dependent, and they increased linearly with age (*p*-value < 0.05; Spearman’s correlation) (Fig. 2D and Supplementary Fig. 3). When splitting the dataset by sex, a positive correlation between age and alpha diversity indices were found for males, but not for females (Fig. 2D and Supplementary Fig. 3). Although both richness and Shannon metrics were noticeably higher for young adult females, no significant results were obtained when comparing males and females aged below 40 years (*p*-value > 0.05; Wilcoxon rank-sum test).

### Gut microbiome associations with diet-related variables

**Figure 3:**
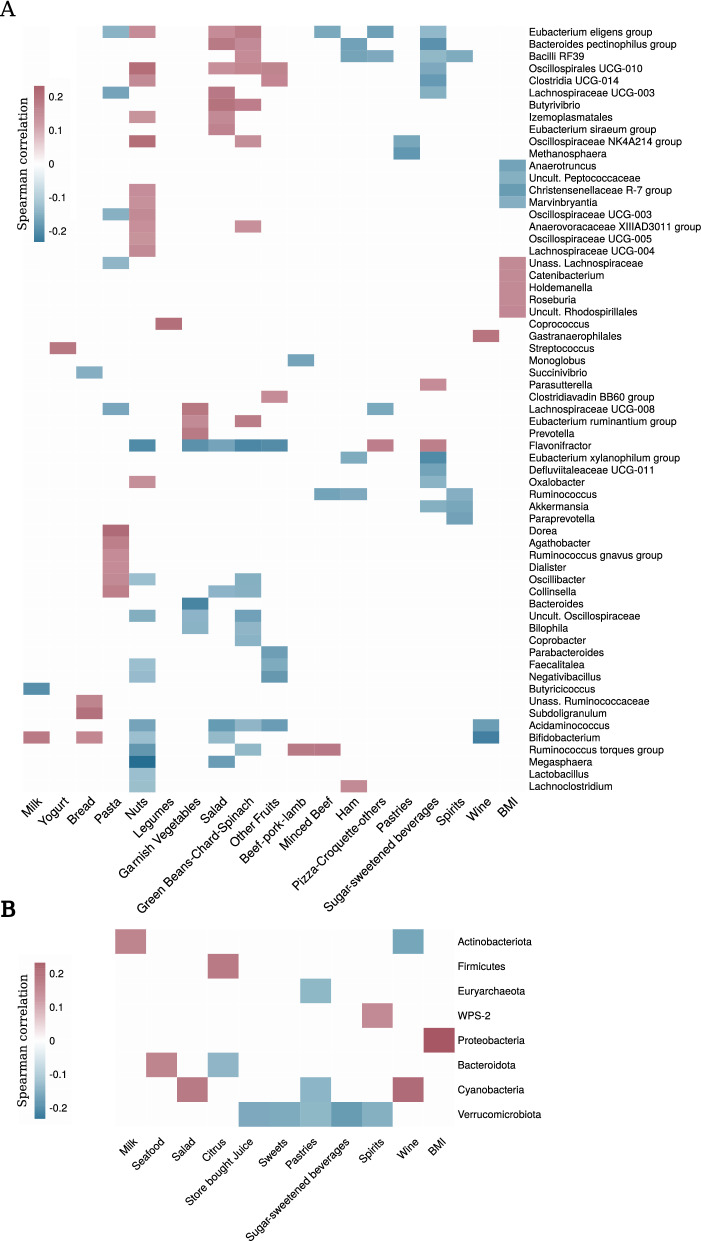
Associations of microbial taxa (**A**: Genera; **B**: Phyla) with the consumption of particular foods or food groups. Only significant associations are shown (FDR-adjusted *p*-value < 0.05). Only taxa or foods/food groups showing at least one significant association are shown. Positive Spearman's rank correlation coefficients (positive associations) are colored in red, while negative values (negative associations) are colored in blue. A full description of the foods included in each food group can be found in the Supplementary Note.

The information on diet habits collected from 528 participants was used to establish correlations between microbiome data (at the phylum and genus level) and the monthly consumption of 43 different foods (e.g., bread, yogurt, milk, etc.) and food groups (e.g., nuts, sugar-sweetened beverages, etc.). Body mass index (BMI) was also included as a variable. In order to reduce the complexity and dimensionality of genus-level microbiome data, only genera with a relative abundance > 0.01% were tested (n = 171). No filtering was applied to the phylum-level data (n = 21). Spearman’s rank correlation was used to identify 63 genera associated with 19 variables (Fig. 3A), and 8 phyla associated with 11 variables (Fig. 3B) (*p*-value < 0.05; Spearman’s rank correlation). *Verrucomicrobiota* and both *Eubacterium eligens* group and *Flavonifractor* resulted in the highest number of significant associations at the phylum and genus level (five and seven associations, respectively). On the other hand, monthly consumption of nuts was associated with the highest number of microbial markers (23 phyla or genera) (Supplementary Table 7).

Although the different diet variables analyzed displayed correlations with different microbial taxa, the consumption of garnish vegetables, salad, green beans/chard/spinach, and nuts, resulted in similar correlations in terms of microbial patterns. Interestingly, these associations tended to be the opposite of those established for the consumption of sugar-sweetened beverages (Fig. 3A).

*Flavonifractor* was a paradigmatic example: it was positively associated with sugar-sweetened drinks, but negatively associated with the consumption of nuts, fruits, and miscellaneous vegetables. Similarly, the abundance of *Ruminococcus torques* group was positively associated with indicators of meat consumption, and negatively associated with nuts and green beans/chard/spinach.

Conversely, *Eubacterium eligens* group was positively associated with nuts, salad and green beans/chard/spinach consumption, and negatively associated with minced beef among others. Remarkably, taxa associated with BMI showed no correlation with other diet variables, except for *Marvinbryantia* and *Christensenellaceae* R-7 group, which were negatively associated with BMI and positively associated with nuts consumption. At the phylum level, a correlation was established between BMI and *Proteobacteria*. Moreover, five diet variables (corresponding to food or food groups with a high sugar content) were associated with a reduction of *Verrucomicrobiota* (Fig. 3B).

Finally, the relationship between age, sex and dietary habits was studied. The monthly consumption of sixteen different foods or food groups was associated to age (Supplementary Table 8), while seventeen diet variables were sex-dependent (Supplementary Table 9). Overall, the consumption of healthier foods (e.g., salad or citrus fruit) increased with age, while less healthy dietary habits (e.g., consumption of sugar-sweetened beverages or chips) presented a negative correlation with this variable. Regarding sex, females tended to adhere to healthier dietary patterns: they reported a lower consumption of beer, spirit drinks or sugar-sweetened beverages, and a higher consumption of fruits and vegetables.

## Discussion

We report here the analysis of the Spanish Microbiome, based on a total of 530 faecal samples, as a proxy of the "normal" gut microbiome of the Spanish population. Although similar studies have been carried out for other European countries, such as UK, Germany, Belgium or the Netherlands^13,18,19,21^, to the best of our knowledge, this is the first large-scale, country-wide gut microbiome analysis in a Mediterranean country.

The two most dominant bacterial phyla in the Spanish gut microbiome were *Firmicutes* and *Bacteroidota,* followed by *Proteobacteria, Verrucomicrobiota* and *Actinobacteriota*. This taxonomic composition is typically reported in healthy individuals^12,19,20,28–31^. The *Firmicutes*/*Bacteroidota* (F/B) ratio has been widely used to detect dysbiosis associated with pathologies, although the usefulness of this marker is currently under debate^32^. Increased or decreased F/B ratio is related to dysbiosis, whereby the former is usually observed with obesity, and the later with inflammatory bowel disease^20,31^. In our study, the average F/B ratio was in line with the ratios observed in other healthy cohorts from distant geographical regions such as the US, UK, Chile, Colombia, and Argentina. However, cohorts from India and Pakistan showed slightly lower F/B values (< 1.0)^32^. In some cases, the F/B ratio can vary by more than an order of magnitude among people^30^, but such large deviations usually are outliers.

At the genus level, the dominant taxa of the Spanish faecal microbiome were *Bacteroides* and *Faecalibacterium*, followed by *Prevotella*, *Alistipes*, and *Oscillospiraceae,* all of them belonging to the dominant phyla *Firmicutes* and *Bacteroidota*. *Bacteroides* and *Faecalibacterium* are typically the dominant genera regardless of the geography of the cohorts^13,19,30,31^. When comparing the relative abundances of *Bacteroides* with previous studies, it should be considered that some members of the former genus *Bacteroides* have recently been reclassified into five genera, i.e., *Alistipes*, *Prevotella*, *Paraprevotella*, *Parabacteroides*, and *Odoribacter*, and hence the relative abundances can vary^33,34^.

In our study, we found minor differences between the microbiome of Spanish males and females. Interestingly, Spanish males showed a higher abundance of *Faecalibacterium* than females. *Faecalibacterium* is a producer of short-chain fatty acids^35^ and one of the most fibrolytic bacteria of the gut^36^. This genus was also found at higher relative abundances in males in other cohorts^37^ and was linked to the development of some metabolic syndromes in men^38^. The differences of some key taxa such as *Faecalibacterium* may contribute to the sexual dimorphism of autoimmune^36,39,40^ and neuroimmune diseases^41^.

The abundance of *Faecalibacterium, Bacteroides, Bifidobacterium* and *Akkermansia* has been proved to depend on age^42^. In our study, a reduction in the abundance of *Faecalibacterium* and *Bifidobacterium* with age was detected, but significant associations between age and *Akkermansia* or *Bacteroides* were not found. *Faecalibacterium* is often described to be negatively associated with age^43–45^, while both negative and positive associations have been found for *Bifidobacterium* and aging^44,46–48^. The abundance of the *Lachnospiraceae* family has also been previously reported to decrease in the elderly^47^. Nonetheless, all the eight taxa belonging to this family (according to SILVA v.138 taxonomy) which displayed a significant association with age in this study (*Lachnospiraceae* UCG-003, *Lachnospiraceae* UCG-008, *Lachnospiraceae* ND3007 group, an unassigned *Lachnospiraceae*, *Butyrivibrio*, *Eubacterium xylanophilum* group, *Eubacterium eligens* group and *Howardella*) showed, indeed, a positive correlation. *Streptococcus* was also negatively associated to age in our dataset, which was in line with previous findings^45^. Apart from the highlighted taxa, the abundance of eighteen additional genera was found to be age-dependent, suggesting that age is a key factor for understanding the gut microbiome of the Spanish population. It has to be noted that taxonomic changes associated with aging may not be directly attributable to age itself, but to different dietary and lifestyle habits across the different age groups^44,49,50^.

Alpha diversity has demonstrated to play an essential role for understanding the human gut microbiome, since loss of microbial diversity have been linked to intestinal dysbiosis in several human diseases^51,52^. With this in mind, investigating the distribution of alpha diversity metrics in the “normal” (or “healthy”) population is key. From all the variables included in the study (sex, BMI, dietary habits, etc.), only age was significantly associated with alpha diversity (positive correlation). This association has previously been found in other European, American and Asian cohorts^53,54^. In fact, according to Badal et al. (2020)^44^ review, no studies have ever confirmed a negative association of alpha diversity with age. Interestingly, our results replicated two specific findings described by de la Cuesta-Zuluaga et al. (2019)^53^: (1) the increase in alpha diversity is more noticeable in young adults, with a change in the trend at around age 40; and (2) young adult females present a higher—yet not significant in this case—alpha diversity (and especially a higher richness) when compared to males of the same age range, suggesting that gut microbiome development throughout the early stages of adulthood may be sex-dependent, although more research is still needed.

Diet has also been proved as a crucial factor for understanding the human gut microbiota configuration and its modulation throughout life^55–57^. It is also worth highlighting that Spain is often considered as a paradigmatic example of adherence to the Mediterranean diet, which has been linked to multiple beneficial effects^58,59^. For that reason, we investigated how the consumption of different foods or food groups influenced the composition of the faecal microbiome.

Some of our findings confirm those of other cohorts and studies, underlining the robustness of the associations across different experimental designs, methodologies and environmental variables. For example, we found a positive correlation between *Bifidobacterium* and dairy intake, as previously reported^60^. Indeed, this association is commonly used as an example of the influence of host genetics in the gut microbiota, since it has been proved that the abundance of *Bifidobacterium* depends on lactase persistence^19,61^. Beyond genetics, a recent study by Asnicar et al. (2021)^45^ has shown a significant positive correlation between lactose and the abundance of three different *Bifidobacterium* species (*B. catenulatum, B. longum* and *B. animalis*). An association between yogurt, galactose and lactose with higher abundances of *Streptococcus thermophilus* was also identified, which matched our observation of a positive correlation between the genus *Streptococcus* and yogurt consumption in the Spanish cohort.

Remarkably, nuts were the food group with the higher number of associations with the microbial markers reported in Asnicar et al. (2021)^45^, as also noted in our study. This could be expected, since nuts are rich in fiber and polyphenols^62^, both of which have been widely demonstrated to modulate the function and composition of the gut microbiota^55,56,63^. Despite discrepancies in the methodologies used in both studies for studying the microbiome (Asnicar et al*.* (2021)^45^ used shotgun metagenomic sequencing and reached the species level, while we used a metataxonomic approach and reached the genus level), some of the identified associations were similar. Specifically, we found nuts consumption to be associated with the *Eubacterium eligens* group, *Bifidobacterium*, and *Flavonifractor*, while Asnicar et al. (2021)^45^ described correlations -in the same direction- for *Eubacterium eligens*, *F. plautii*, *B. bifidum* and *B. catenulatum,* but the opposite correlation was found for *B. animalis*. We identified a negative correlation for the abundance of *Lactobacillus* in the gut and nut consumption. Similarly, Creedon et al*.* (2020)^62^ described that pistachio consumption decreased the number of lactic acid bacteria. Paradoxically, although nuts have proved to have potential prebiotic effects^64,65^, negative correlations between nut consumption and *Lactobacillus* and *Bifidobacterium,* which are commonly used as probiotics, have been established. So, further research is still needed in order to depict the underlying mechanisms explaining this phenomenon.

We also revealed microbial associations in opposite directions between food groups that usually reflect antagonistic dietary habits (i.e., nuts, vegetables and fruits vs. meat and sugary drinks), which was in line with the behaviour described by Asnicar et al*.* (2021)^45^ For instance, *Flavonifractor* showed a negative correlation with healthy plant-based food groups (garnish vegetables, salad, green beans/chard/spinach, other fruits and nuts), and a positive correlation with less healthy food groups (sugar sweetened beverages and the group formed by pizza, croquette and others). Surprisingly, these results overlapped almost completely with the associations described for *F. plautii* by Asnicar et al*.* (2021)^45^, which indeed reported negative correlations between this species and multiple health indicators, including BMI and visceral fat. The genus *Flavonifractor* and the species *F. plautii* have been previosuly linked to different diseases and disorders, such as major depression disorder^66^, bipolar disorder^67^ or colorectal cancer^68^. Moreover, it is worth highlighting that *F. plautii* was negatively associated with adherence to MD^69^, while its abundance was reduced after switching to this diet^70^. *F. plautii* is a flavonoid-degrading bacterium, which has been suggested to potentially reduce the bioavailability of beneficial flavonoid^67,68^, although the specific mechanisms explaining the role of *Flavonifractor* in health and disease are not yet clear and require further investigation.

Another interesting case is that of *Eubacterium eligens* group and *Ruminococcus torques* group. The former was positively associated with healthy foods and negatively associated with minced beef (including hamburgers and sausages), spirits and the pizza/croquette/other group, which was similar to what was reported by other studies^18,45^. The latter was rather positively and negatively associated to animal-based and plant-based foods, respectively. These bacteria have been previously linked to vegetarian (*Eubacterium eligens*) and non-vegetarian (*Ruminococcus torques*) subjects^71^. Moreover, *Eubacterium eligens* was linked to a higher adherence to the MD^69^ and to a beneficial health signature^45^, while *Ruminococcus torques* has been negatively associated with MD^69,70^. Altogether, and considering that the Spanish population mainly follow the MD, *Flavonifractor* (or *F. plautii*), *Ruminococcus torques* group, and *Eubacterium eligens* group could be proposed as gut microbial markers of adherence to this diet.

Other remarkable associations found in the studied Spanish cohort were:

1. a negative correlation between *Akkermansia*, a genus which has been inversely linked to several disease states^72^ and higher consumption of sugary drinks and spirits. This contrasted with previous studies^73,74^, although a negative association between *Akkermansia* and other sugar-rich foods has been previously reported^75^.
2. An inverse correlation between wine consumption and both *Bifidobacterium* and *Acidaminicoccus*. Interestingly, a decrease in the abundance of *Acidaminococcus* sp. D21 has been linked to alcohol dependence syndrome and liver cirrhosis^76^, while a negative correlation between *B. bifidum* and alcohol intake has been previously described^45^.
3. A positive correlation between legumes consumption, one of the key signatures of MD, and *Coprococcus.* This genus proved to increase with higher quality of life indicators and to be depleted in depression^77^, but its interrelationship with dietary habits is yet unclear.
4. Several correlations between BMI and different genera, including a positive association with the abundance of *Roseburia.* A positive correlation between *R. inulinivorans* and BMI was also described by Asnicar et al*.* (2021)^45^, although other members of the *Roseburia* genus (i.e., *R. hominis* or *Roseburia* bacterium CAG:182) have been linked to healthy plant-based foods, adherence to MD and other indicators of good health^45,69,70^. This suggests a species-dependant behaviour of the associations between *Roseburia* and health parameters, including BMI.

Overall, the present work constitutes the first complete analysis of the gut microbiome of a Mediterranean country. Our findings clearly confirm the association between some of the foods that characterize the Mediterranean diet (vegetables and nuts, basically) with the abundance of bacterial taxa which, in turn, are associated with health benefits. Moreover, dietary habits have demonstrated to be sex- and age-dependent. In particular, females tended to adhere to healthier dietary patterns, while young individuals reported an increased consumption of less healthy foods or food groups. This suggests that adherence to MD in Spain is decreasing among young people, which could have a negative impact on the health condition and gut microbiome composition of the Spanish population. Despite the difficulties encountered when comparing our investigation with other studies because of methodological bias, our results demonstrate or point out the robustness of the associations between healthy lifestyle traits and particular microbial markers, some of which have not been deeply studied in previous nation-wide microbiome studies. Taken together, our results strongly suggest a double effect of the Mediterranean diet on the health of the Spaniards: direct ones (i.e. “inherent” nutritional values such as content in vitamins, fiber, healthy fats, etc.) and indirect ones through a not-yet fully understood, but yet strong association between specific foods, microbial markers and health.

## Materials and methods

### Study design, participant recruitment, and sample collection

This study targeted healthy citizens with an age ranging from 18 to 70 years, who did not meet any of the exclusion criteria summarized in the Supplementary Note. Briefly, the main exclusion criteria were related to a range of infections, diseases/syndromes, as well as to the intake of antibiotics in the last 6 months. A sample group of 530 individuals was designed in accordance with official demographic data (www.ine.es) in order to have a representative picture of the Spanish population in terms of age, sex, and geography (distributed in 17 autonomous regions and 2 autonomous cities). The number of individuals corresponding to each criterium is shown in Supplementary Table 1.

Volunteer participants were recruited through the ICLANP website (https://ienp.es/), and selected as follows: first, a standard EQ-5D-5L questionnaire (EuroQol Group, 2009) (Supplementary Note) was used to evaluate the generic health status and to pre-select participants with satisfactory values in five different aspects (mobility, self-care, usual activities, pain/discomfort, and anxiety/depression). Second, an ad hoc questionnaire was used to retrieve participant’s data regarding age, sex, and location, and to evaluate the different exclusion criteria. Finally, the volunteers that obtained a positive evaluation in the previous steps, completed an additional online questionnaire regarding dietary habits (Supplementary Note). Specifically, participants were asked for their weekly and/or monthly consumption (in number of portions) of 43 different foods (e.g., bread, yogurt, milk, etc.) and food groups (e.g., nuts, sugar-sweetened beverages, etc.) (Supplementary Table 2). For those participants that only provided the weekly consumption of certain foods or food groups, the monthly consumption was calculated by multiplying the weekly consumption by four. This data was used for establishing associations between diet-related variables and microbial markers, as described below.

The selected participants signed an informed consent containing all the information on the objectives, general methodology, and data management and protection aspects of the study. Stool sampling was performed by each participant with the Stool Nucleic Acid Collection and Preservation Kit (Norgen Biotek), and the anonymized samples were processed at the facilities of Darwin Bioprospecting Excellence, SL (Valencia, Spain) following the IHMS (International Human Microbiome Standards).

### Microbiome sequencing

Samples were received and processed in different batches from May 2019 to September 2020. Briefly, DNA was extracted from stool samples using the Stool Nucleic Acid Isolation Kit (Norgen Biotek) following the manufacturer’s instructions. Then, Qubit × 1 dsDNA HS Assay kit (Qubit 2.0 Fluorometer, Thermo Fisher, Waltham, United States) was used for DNA quantification. The amplicon sequencing protocol targeted the V3 and V4 regions (459 bp) of the 16S rRNA genes with the primers designed on surrounding conserved regions^78^. Following the Illumina amplicon libraries protocol, DNA amplicon libraries were generated using a limited cycle PCR: initial denaturation at 95 °C for 3 min, followed by 25 cycles of annealing (95 °C 30 s, 55 °C 30 s, 72 °C 30 s) extension at 72 °C 5 min, using a KAPA HiFi HotStart ReadyMix (KK2602). Then, Illumina sequencing adaptors and dual-index barcodes (Nextera XT index kit v2, FC-131–2001) were added to the amplicons. Libraries were normalized and pooled prior to sequencing. The pool containing indexed amplicons was then loaded onto the MiSeq reagent cartridge v3 (MS-102–3003) spiked with 10% PhiX control to improve base calling during sequencing, as recommended by Illumina for amplicon sequencing. Sequencing was conducted using a paired-end, 2X250pb and 2 × 300pb cycle runs on an Illumina MiSeq device.

### Bioinformatic and statistical analysis

Raw Illumina sequences were analysed using Qiime2 (v. 2020.8)^79^. Briefly, the quality of the reads was assessed with the Demux plugin, and primers were subsequently trimmed from the beginning of the reads via DADA2 (q2‐dada2)^80^. This plugin was also used for denoising and truncating the reads at 245 bp, and for clustering them into amplicon sequence variants (ASVs). The taxonomy of each sequence variant was assigned employing the q2‐feature‐classifier classify‐sklearn naïve Bayes taxonomy classifier^81^. SILVA (v. 138)^82^ was used as reference database for taxonomic assignment. SILVA taxonomy nomenclature was used for preparing the figures (i.e., *Bacteroidota* was used instead of *Bacteroidetes*).

Microbiome data was analyzed and visualized in R, mainly using phyloseq^83^ and ggplot2 packages. Rarefaction curves were constructed with the iNEXT and ggiNEXT functions (iNEXT)^84^. Correlations between quantitative variables (i.e., monthly consumption of foods or food groups, BMI, age, etc.) and the relative abundance of microbiome markers were evaluated using Spearman’s rank correlation test. Categorical variables (i.e., sex, region, etc.) were tested with DESeq2, using its built-in function for normalizing the raw microbiome counts^85^. All the *p*-values obtained in both cases were adjusted using *p*. adjust R function with the Benjamini–Hochberg procedure (FDR), and significance was defined at adjusted *p*-value < 0.05. Significant correlations were plotted into heatmaps using pheatmap package. Alpha diversity metrics (richness and Shannon) were calculated at the ASV level. For alpha diversity tests, all the samples were rarefied to 15.000 reads to mitigate uneven sequencing depth. Seventeen samples did not reach this threshold and, therefore, were discarded for alpha diversity analysis.

### Ethics approval

All the proceedings followed in this study (including data management and protection) were approved by the Ethics Committee of Hospital Clínico San Carlos (Madrid, Spain; project reference 18/557-E). All methods were performed in accordance with the relevant guidelines and regulations. All the participants of the study gave their informed consent.

## Supplementary Information

Supplementary Information 1.

Supplementary Figures.

Supplementary Table 1.

Supplementary Table 2.

Supplementary Table 3.

Supplementary Table 4.

Supplementary Table 5.

Supplementary Table 6.

Supplementary Table 7.

Supplementary Table 8.

Supplementary Table 9.

## Data Availability

Raw microbiome sequencing data is available for research use only. Access to the data can be requested using the following online form: https://forms.office.com/r/v8rG8j6r0B. Supplementary Table 2 includes the metadata of the study. The code and intermediate files used for analyzing the data and creating the figures have been deposited on Github (https://github.com/adlape95/ME). The authors confirm that the rest of the results have been included in the article and/or uploaded as supplementary material.
