# Supplementary material for: The Spanish gut microbiome reveals links between microorganisms and Mediterranean diet
Source: Sci Rep. 2021 Nov 10;11:21602. doi: 10.1038/s41598-021-01002-1 (PMC8580991; doi:10.1038/s41598-021-01002-1)
Supplement: Supplementary file 1 — Supplementary Information 1. [file 41598_2021_1002_MOESM1_ESM.pdf]

SUPPLEMENTARY NOTE

The Spanish gut microbiome reveals links between microorganisms and Mediterranean Diet

Adriel Latorre-Pérez[a*], Marta Hernández[b*], Jose Ramón Iglesias[b], Javier Morán[c], Javier Pascual[a], Manuel Porcar[a,d], Cristina Vilanova[a] & Luis Collado[e]

[a] *Darwin Bioprospecting Excellence S.L., Parc Científic Universitat de València, Paterna, Spain*
[b] *Instituto Central Lechera Asturiana de Nutrición Personalizada (ICLANP), Siero, Spain.*
[c] *Instituto de Innovación Alimentaria. Universidad Católica de Murcia, Spain*
[d] *Institute for Integrative Systems Biology I2SysBio (University of València-CSIC), Paterna, Spain*
[e] *Department of Medicine, Complutense University of Madrid, Madrid, Spain*

* Corresponding authors:

Marta Hernández, Instituto Central Lechera Asturiana de Nutrición Personalizada, Siero, Spain; marta.hernandez@capsa.es

Adriel Latorre-Pérez, Darwin Bioprospecting Excellence S.L., Paterna, Spain; alatorre@darwinbioprospecting.com

INCLUSION/EXCLUSION CRITERIA

Inclusion criteria:

• Male or female subjects

• Age between 18 and 70 years at the time of inclusion in the study.

• Must be able to provide signed and dated informed consent.

• Healthy subjects willing and able to provide stool samples.

Exclusion criteria:

Any subject who meets any of the following criteria was excluded from participation in this study:

• Body mass index greater than or equal to 35 or less than or equal to 18 at the time of inclusion in the study.

• Use of any of the following medications in the past 6 months:

- Systemic antibiotics (intravenous, intramuscular or oral); Oral, intravenous, intramuscular, nasal or inhaled corticosteroids; Cytokines

- Methotrexate or immunosuppressive cytotoxic agents

- Large doses of commercial probiotics consumed (greater than or equal to 108 CFU / day): include tablets, capsules, lozenges, gum or powders in which the probiotic is a primary component. Ordinary dietary components such as fermented drinks / milks, yogurts, food do not apply)

• Acute illness at the time of enrollment. Acute illness is defined as the presence of moderate or severe

illness with or without fever.

• Chronic, clinically significant (unresolved, requiring ongoing medical treatment or medications) lung, cardiovascular, gastrointestinal, liver, or kidney function abnormalities, as determined by medical history.

• History of cancer, except squamous or basal cell carcinomas of the skin that have been managed medically by local excision.

• The unstable dietary history defined by major changes in the diet during the previous month, where the subject has eliminated or significantly increased an important food group in the diet.

• Recent history of chronic alcohol use.

• Positive test for HIV, HBV or HCV.

• Any confirmed or suspected immunosuppression or immunodeficiency condition / status (primary or acquired)

• Major surgery of the gastrointestinal tract, with the exception of cholecystectomy and appendectomy, in the last five years. Any major bowel resection at any time.

• History of active uncontrolled gastrointestinal disorders or diseases including: inflammatory bowel disease (mild-moderate-severe ulcerative colitis, mild-moderate-severe Crohn's disease, or indeterminate colitis); moderate-severe irritable bowel syndrome; persistent and infectious gastroenteritis, colitis or gastritis, persistent or chronic diarrhea of unknown etiology, recurrent *Clostridium difficile* infection or untreated *Helicobacter pylori* infection; chronic constipation.

• Pregnant or nursing women.

- Treatment or suspicion of having suffered toxic shock syndrome.

- Patients who cannot understand or complete the proposed questionnaires.

DIETARY HABITS QUESTIONNAIRE.

Indicate the number of times you eat the following products per week and per month:

- Milk
- Yogurt
- Chocolate: tablet, chocolates, "Kit Kat", "Mars", etc.
- Puffed breakfast cereals (“Corn-Flakes”, “Kellog’s”)
- Buscuits
- Chocolate/caramel cookies
- Pastries: donut, croissant, etc.
- Salad: lettuce, tomato, escarole, etc.
- Green beans, chard, or spinach
- Garnish vegetables: aubergine, mushrooms and vegetables that have not been included in any other category.
- Baked, fried or boiled potatoes
- Legumes: lentils, chickpeas, beans, etc.
- White rice or paella
- Pasta: noodles, macaroni, spaghetti, etc.
- Soups and creams
- Eggs
- Chicken or turkey
- Beef, pork, lamb (steak, pie, etc.)
- Minced meat, sausage, hamburger, meatball
- White fish: hake, grouper, etc.
- Blue fish: sardines, tuna, salmon, etc.
- Seafood: mussels, prawns, prawns, squid, etc.

- Croquettes, dumplings, pizza
- Bread (in a sandwich, with meals, etc.)
- Ham: Cured ham, boiled ham, sausages
- White or fresh cheese or low in calories
- Other cheeses: cured or semi-cured, creamy
- Citrus fruits: orange, tangerine, etc.
- Other fruits: apple, pear, peach, banana, etc.
- Canned fruits (i.e., in syrup)
- Natural fruit juices
- Store bought Juice
- Nuts: peanuts, hazelnuts, almonds, etc.
- Dairy desserts: custard, flan, cottage cheese
- Cream or chocolate cakes
- Sweets: gummies, candiesm etc.
- Snack bags ("chips", "chetos", "fried")
- Ice creams
- Sugar-sweetened beverages ("Coke", "Fanta", “Sprite”, etc.)
- Low calorie drinks (Diet Coke...)
- Wine (or sangria)
- Beer
- Alcohol-free beer
- Spirits: whiskey, gin, rum, etc

Health Questionnaire

English version for the UK

Sample

UK (English) © 2009 EuroQol Group EQ-5D™ is a trade mark of the EuroQol Group

Under each heading, please tick the ONE box that best describes your health TODAY.

MOBILITY

I have no problems in walking about ❑

I have slight problems in walking about ❑

I have moderate problems in walking about ❑

I have severe problems in walking about ❑

I am unable to walk about ❑

SELF-CARE

I have no problems washing or dressing myself ❑

I have slight problems washing or dressing myself ❑

I have moderate problems washing or dressing myself ❑

I have severe problems washing or dressing myself ❑

I am unable to wash or dress myself ❑

USUAL ACTIVITIES *(e.g. work, study, housework, family or leisure activities)*

I have no problems doing my usual activities ❑

I have slight problems doing my usual activities ❑

I have moderate problems doing my usual activities ❑

I have severe problems doing my usual activities ❑

I am unable to do my usual activities ❑

PAIN / DISCOMFORT

I have no pain or discomfort ❑

I have slight pain or discomfort ❑

I have moderate pain or discomfort ❑

I have severe pain or discomfort ❑

I have extreme pain or discomfort ❑

ANXIETY / DEPRESSION

I am not anxious or depressed ❑

I am slightly anxious or depressed ❑

I am moderately anxious or depressed ❑

I am severely anxious or depressed ❑

I am extremely anxious or depressed ❑

Sample

UK (English) © 2009 EuroQol Group EQ-5D™ is a trade mark of the EuroQol Group

- We would like to know how good or bad your health is TODAY.
- This scale is numbered from 0 to 100.
- 100 means the best health you can imagine.
  0 means the worst health you can imagine.
- Mark an X on the scale to indicate how your health is TODAY.
- Now, please write the number you marked on the scale in the box below.

YOUR HEALTH TODAY = ☐

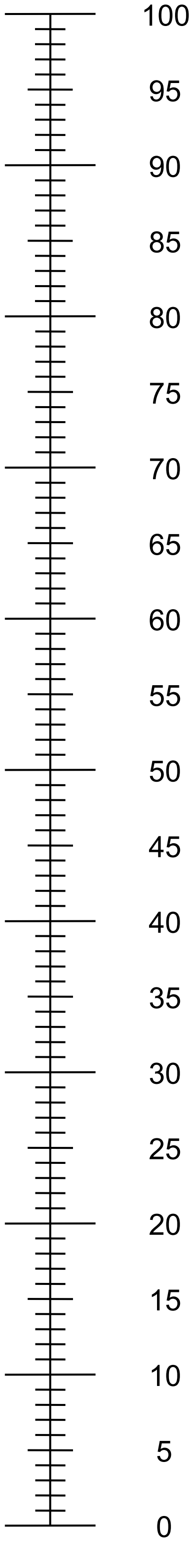

Sample

UK (English) © 2009 EuroQol Group EQ-5D™ is a trade mark of the EuroQol Group
