# Supplementary material for: The Spanish gut microbiome reveals links between microorganisms and Mediterranean diet
Source: Sci Rep. 2021 Nov 10;11:21602. doi: 10.1038/s41598-021-01002-1 (PMC8580991; doi:10.1038/s41598-021-01002-1)
Supplement: Supplementary file 2 — Supplementary Figures. [file 41598_2021_1002_MOESM2_ESM.pdf]

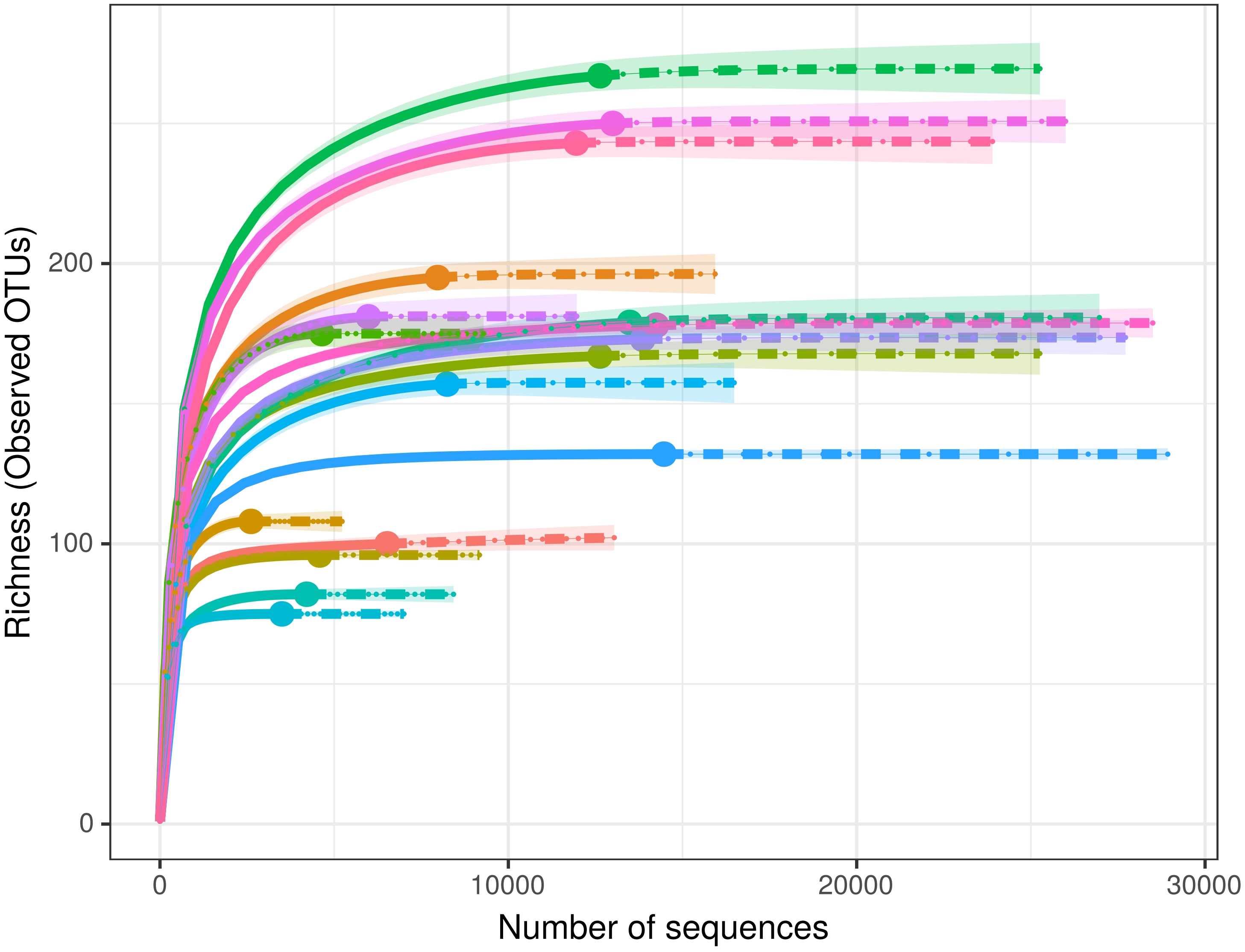

Supplementary Figure 1. Rarefaction curves. Only samples with <15K reads are shown for clarity. The rest of samples, which had more than 15K reads per sample, presented similar rarefaction curves.

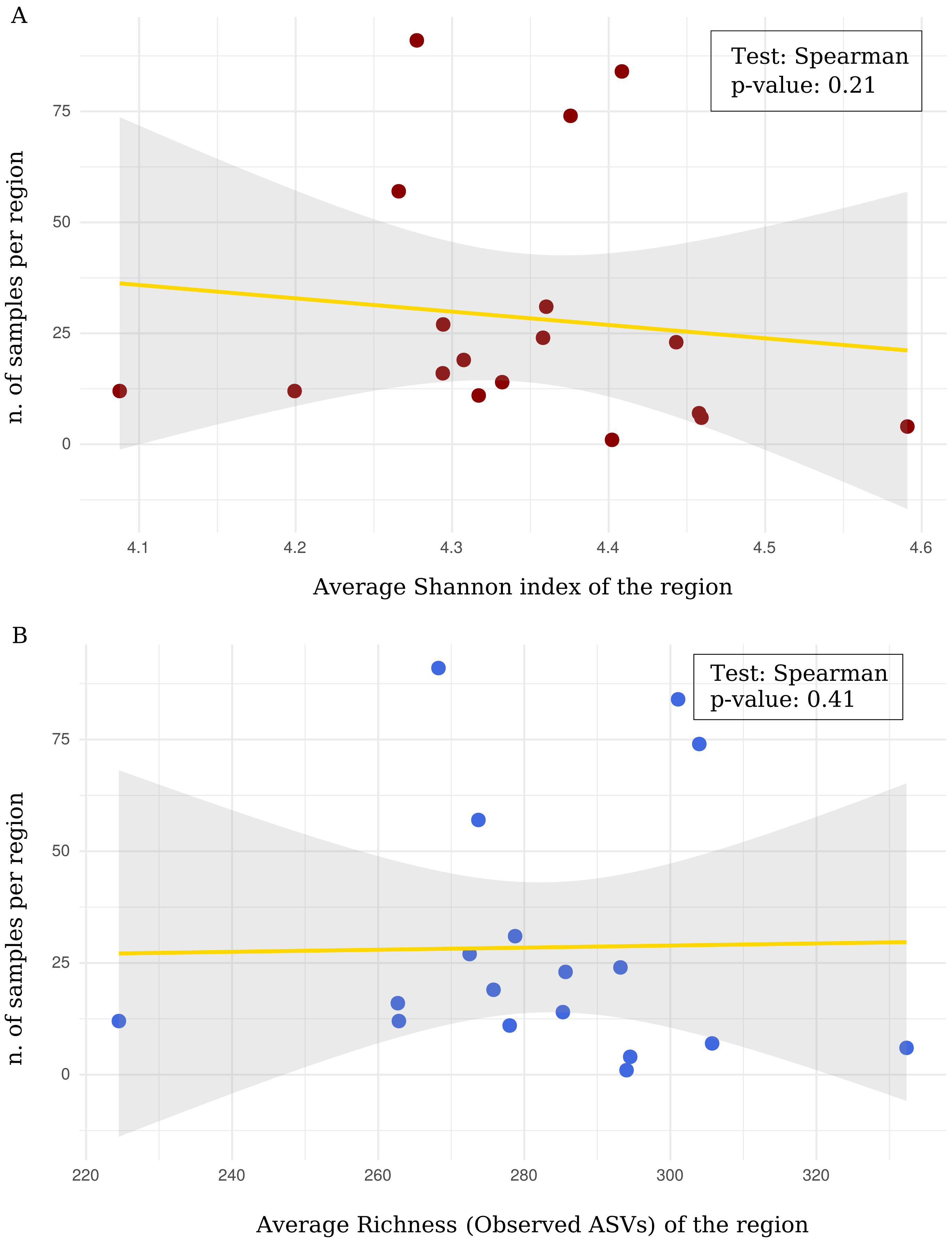

Supplementary Figure 2. Shannon (A) and richness (B) indices vs. number of samples for each region. There is not a significant association between the number of individuals recruited and the average alpha diversity measures obtained for each region.

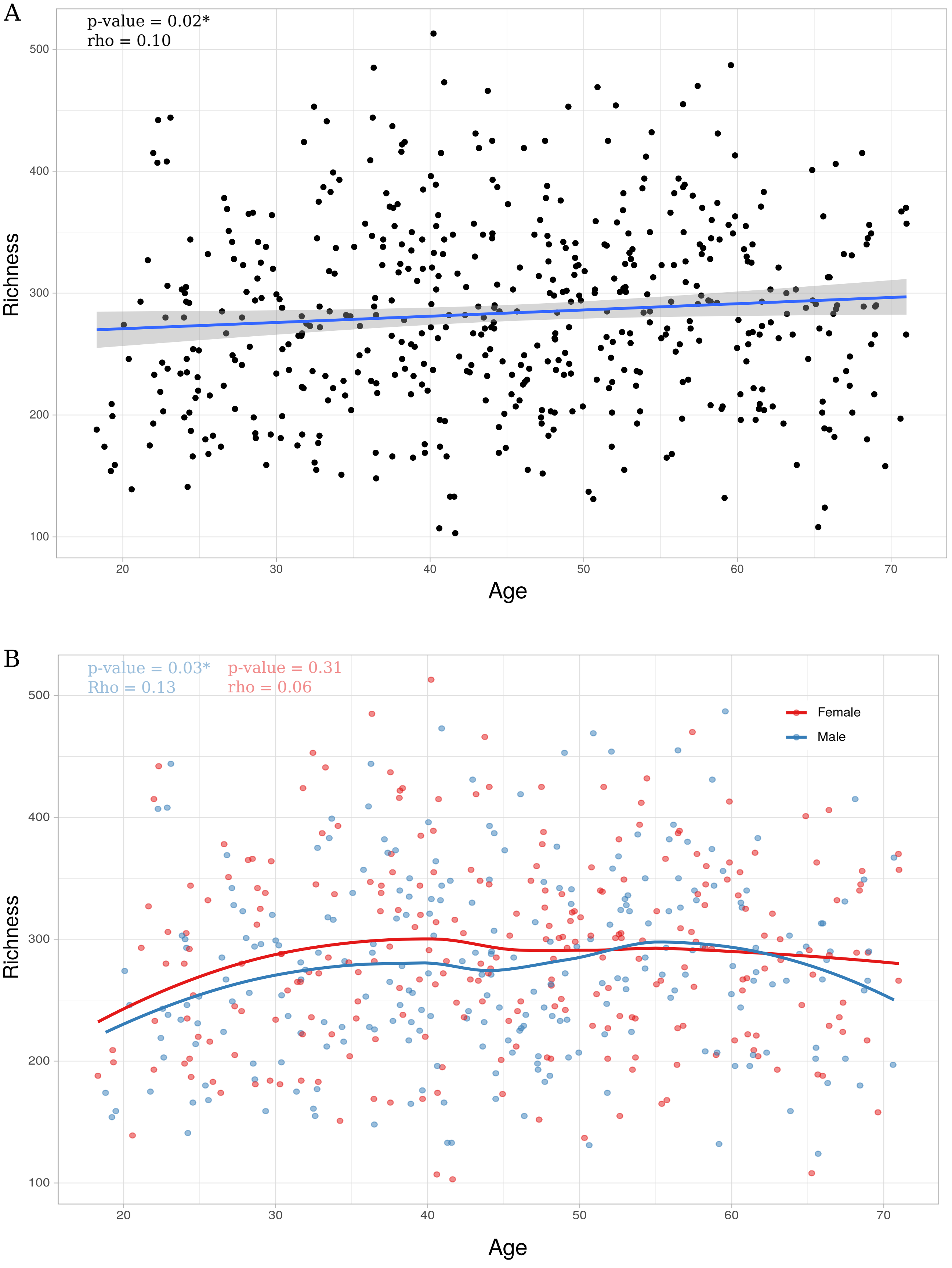

Supplementary Figure 3. Richness (observed ASVs) vs. age considering the whole cohort (A) (linear model "lm" used as smoothing method) or separating by sex (B) (local polynomial regression fitting model "loess" used as smoothing method). Rho and p-values resulting from Spearman correlation are shown. Black = both females and males; Red = females; Blue = males.
